# Data on nearly zero energy buildings (NZEBs) projects and best practices in Europe

**DOI:** 10.1016/j.dib.2021.107641

**Published:** 2021-11-26

**Authors:** Delia D'Agostino, Sofia Tsemekidi Tzeiranaki, Paolo Zangheri, Paolo Bertoldi

**Affiliations:** a European Commission, Joint Research Centre (JRC), Ispra, VA, Italy; b Enea, Ispra, VA, Italy

**Keywords:** European energy policy, Nearly zero energy buildings (NZEBs), Technologies, Energy efficiency in buildings, Renewable sources, Costs, NZEBs, Nearly zero energy buildings, MS, Member States, EU, European Union, EPBD, Energy Performance of Building Directive, EPC, Energy performance certificate, LTRS, Long term renovation strategy, RES, Renewable Energy Sources, PED, Primary Energy Demand, c-o, Cost-optimal, AT, Austria, BE-BRU, Belgium-Brussels region, BE-FLA, Belgium- Flemish region, BE-WA, Belgium- Wallonia, BG, Bulgaria, CY, Cyprus, CZ, Czech Republic, DE, Germany, DK, Denmark, EE, Estonia, EL, Greece, ES, Spain, FI, Finland, FR, France, HR, Croatia, HU, Hungary, IE, Ireland, IT, Italy, LT, Lithuania, LU, Luxemburg, LV, Latvia, MT, Malta, NL, Netherlands, PL, Poland, PT, Portugal, RO, Romania, SE, Sweden, SI, Slovenia, SK, Slovakia, UK, United Kingdom

## Abstract

This data article refers to the paper “Assessing Nearly zero energy buildings (NZEBs) development in Europe” [1]. Data linked with this article relate to collected best practices NZEBs throughout Europe. Data on building geometry, year of construction or renovation, primary energy consumption, saving percentages, renewable production, heating demand are provided.

Data allow an overview of the status of most commonly implemented efficiency measures and renewables in NZEBs. In particular, data are available in relation technologies, such as heating, domestic hot water, lighting, renewable sources, ventilation, cooling. Heat recovery efficiency data are also collected. U-values are detailed for roofs, walls, floors, windows. Further data can be visualized in relation to technologies costs, cost of construction and maintenance.

## Specifications Table

**Table:** 

Subject	Energy
Specific subject area	Building data, energy consumption, energy efficiency measures.
Type of data	Data in spreadsheet format (.xlsx).
How the data were acquired	Data collected from different literature sources, mainly research projects on NZEBs.
Data format	Raw data for which consistency was checked among Member States.
Parameters for data collection	Data were acquired collecting information on NZEB buildings/projects.
Description of data collection	We selected the datasets considering the most recent recognized projects containing data on specific aspects (e.g. technologies, costs) on NZEBs at European level. Data on NZEBs best practices relate implemented technologies and systems used for heating, cooling, ventilation, lighting, domestic hot water and renewables. In addition, data for the energy needs as well as the renewable contribution have been collected and available within this paper to visualize different information on NZEBs. Other data are related to installation and maintenance costs.
Data source location	Data are taken from [2], [3], [4], [5], [6], [7], [8], [9].
Data accessibility	Data are provided in supplementary materials directly with this article.
Related research article	D. D'Agostino, S. Tsemekidi Tzeiranaki, P. Zangheri, P. Bertoldi, Assessing Nearly Zero Energy Buildings (NZEBs) development in Europe, Energy Strategy Reviews 36 (2021) 100680, 10.1016/j.esr.2021.100680

## Value of the Data

• The data are useful to follow the implementation of NZEBs and related technological measures.
• The data can be used to have quantitative information on NZEBs in Europe in terms of energy consumption, efficiency measures and costs.
• The data give insight on retrofit solutions for envelope, appliances, and systems in NZEBs.
• The data support energy efficiency and energy policies related to buildings.
• The data can be useful for the development of NZEBs, comparison with other building types, retrofit intervention, or further analysis.

## 1 Data Description

Buildings are of strategic importance of European policies aimed at limiting greenhouse gas emissions [10]. Although European policies encouraged the construction sector to move towards NZEBs [11], the majority of NZEBs are still demonstration projects, indicating that a full implementation of the concept is not yet reached [12]. Identifying best practices help the NZEBs diffusion. Reported data relate to NZEBs projects and best practices in European Member States as collected from different sources [2], [3], [4], [5], [6], [7], [8], [9].

Data on NZEBs best practices are attached to this paper in the form of an excel spreadsheet (named “NZEBs”). It is composed of different sheets:

In Sheet 1 (named “NZEBs best practices”), the following information is available for each collected best practice building:

• Building/project name and category (e.g. office, hotel)
• Member State location (country in which the building is located)
• Year of construction or refurbishment (year in which the building was constructed or renovated)

Implemented technologies (systems installed in the building to cover the energy needs, e.g. heat pumps)

• Primary Energy Demand (PED) (kWh/m^2^y) (amount of energy that must be generated to satisfy the total energy demand of the building)
• Renewables (RES) (%) (percentage of renewable energy in the building)
• Floor area (m^2^) (area of the building)
• Space heating demand (kWh/m^2^y) (amount of heating required to heat the building)
• Building Type (e.g. residential, non-residential)

In Sheet 2 (named “Technologies”):

• Building/project name
• Member State location
• Year of construction and refurbishment
• Building type
• Implemented technologies (e.g. gas boiler, district/decentralized/heating/cooling, mechanical ventilation with heat recovery, biomass, PV, solar thermal, heat pump, rainwater/lakewater, water pump, floor heating, heat exchanger, geothermal, energy saving/intelligent lamps, CHP, wind turbine, natural cooling/ ventilation)

In Sheet 3 (named “Technology cost”):

• Implemented technology
• Member State location
• Installation (cost to install the technology)
• Grid connection (in relation to photovoltaic systems)
• Product cost (cost to have the technology)
• Initial design cost (cost of the initial design of the technology)
• Operational energy use revenues (from electricity sold to the grid)
• Trends/objectives (projection trends or objective related to the technology, if available)

In Sheet 4 (named “Cost”):

• Building/project name
• Member State location
• Construction cost (€/m^2^) (cost to build the building)
• Average construction cost per Member State (€)
• Lifecycle maintenance cost (€/m^2^) (cost to maintain the building over its lifecycle)
• Average lifecycle maintenance cost per Member State
• Data source (where the information was taken)

In Sheet 5 (named “Energy consumption-Renewables”):

• Building name/project
• Member State location;
• Renewable (%)
• Primary Energy Demand (kWh/m^2^y)
• Space heating Demand (kWh/m^2^y)
• Average RES share in best practice example buildings by MS (average of the renewable energy present in the buildings)
• Average primary energy demand in best practice example buildings by MS (average of the primary energy demand)
• Average space heating demand in best practice example buildings by MS (average of the space heating demand)
• Data source

In Sheet 6 (named “Heat recovery”):

• Building/project name
• Member State location;
• Heat Recovery Efficiency (efficiency of the installed heat recovery system)
• Average heat recovery efficiency per MS (average of the above)
• Data source

In Sheet 7 (named “Systems”):

• Implemented technologies
• Number of best practice buildings where a technology was installed (buildings having a certain technology)
• Relative percentage (derived percentage of the building having a certain technology)

In Sheet 8 (named “U-values”):

• Building/project name
• Member State location
• Data source
• U-value for walls, roofs, floors, windows (or heat transfer coefficient: measure of heat loss through a building shell element, e.g. walls, roofs, floors, windows)
• Average U-Value for NZEB exemplary buildings per MS (average value of the above)

## 2 Experimental Design, Materials and Methods

The 51 buildings included in the data are located in 20 Member States (BE, FR, IT, IE, AT, SE, UK, EE, DE, FI, DK, SI, NL, BG, HR, PL, MT, PL, LU, HU). Italy and Austria represent the largest part of NZEBs of the sample (12% each), followed by France and Germany (10% each). Various types of buildings are included in the sample, like educational, commercial, office and residential buildings from 19 Member States. The 43% of the buildings are educational buildings (schools, nurseries, child-care facilities, universities). Some of them are pilot projects. The 33% are residential buildings (family houses, apartments). The 12% are office/administration buildings. Finally, there are also hotels and mixed used urban projects. The building/projects included have been constructed during the period from 2002 to 2019. However, for some of them the construction year is not available.

**Fig. 1:**
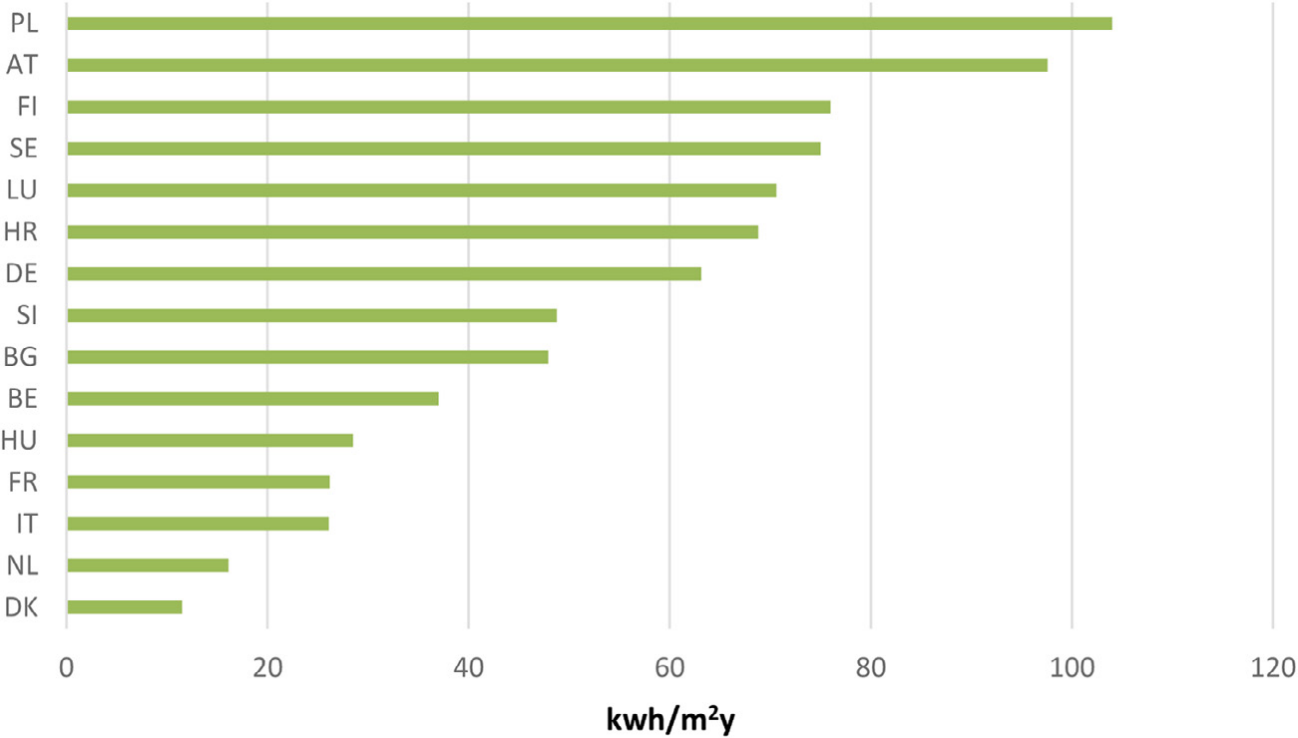
Average primary energy demand and heating demand in best practice example buildings by MS [2], [3], [4], [5], [6], [7], [8], [9].

Data allow visualize the average primary energy demand values (including renewables) of NZEBs best practices per country, as shown in Fig. 1.

In the collected NZEBs best practices, the projects with available on energy demand data are 28, ranging from the *École François Mitterand* in France (−7.5 kWh/m^2^y) to the *Plus Energy Settlement in Kleehäuser*, Freiburg in Germany (152 kWh/m^2^y) (2006) [6]. Data are higher for Member States with colder climate. An exception to this is the Green Lighthouse in Denmark (3 kWh/m^2^y) [2]. Some variations to this pattern may be explained by the differences in construction years and consequently in legislation and technology development.

Data allow also visualise the most diffused NZEBs technologies. As example, a NZEB best practice building using geothermal heat pumps for heating is the *Primary School in Bielawa* in Poland. An example of NZEB using ambient air heat pumps for heating is the *Technical University Sofia* in Bulgaria. Finally, an example of a building using water heat pumps is the *Faculty of Agriculture, University of Osijek* in Croatia [2,3]. Reported energy services differ within the collected building sample. As example, heating is available in all buildings (e.g. solar thermal, gas and pellet boiler, heat and water pump, district, cogeneration, and floor heating), cooling (natural and mechanical) is reported in 17 buildings, and domestic hot water (e.g. heat pump, solar thermal, district, boiler) in 36.

Main costs associated with technologies are available. As example, Table 1 is obtainable from the data linked to this paper [5]. It summarizes the main costs associated to heat pumps in 4 Member States (France, Bulgaria, Spain and Italy). Total cost includes installation cost, product cost, initial design cost, annual maintenance cost as well as operational energy costs. Same kind of tables can be obtainable for other technologies from the provided data.

**Table 1:** Cost associated to Heat Pump technology in France, Bulgaria, Spain and Italy [5].

	Installation	Product cost	Initial design cost	Annual maintenance cost	Operational energy costs	Trends/objectives
FR	485-2,100 €	2,200-13,000 €		150-200 €	14, 16 c€/kWh and 100 €/year fixed cost for 6 kW	No specific objective regarding heat pumps
BG	2,000 €	11,000 €	500 €	300 €		Steady development and increase in the number of installations
ES	700-7,000 full installed	178-826 €/kW installed		14,5-800 /kW installed	0,117 €/KWh	No specific objectives for the heat pumps, except for geothermal heat pumps, which have specific financial aid
IT	13-60 €/kW	130-700 €/kW	10% (on the whole plant system: generation, emission, distribution, controls).		Electricity prices: 0,13 €/kWh + 10 €/month (VAT excluded)15Gas prices:0.091 €/kWh	50-65% tax incentives for heat pumps

Data are also collected for specific technologies. For boilers, there are NZEBs pilot education buildings in Europe using this technology for space heating [2,3]. Examples are the *École François Mitterand in* and the *Hill Primary School* in the United Kingdom. For biomass boilers, *Elementary School and Kinder-garten Albrechtsberg* in Austria is a best practice example of a pilot education building using biomass boiler for space heating and domestic hot water.

**Fig. 2:**
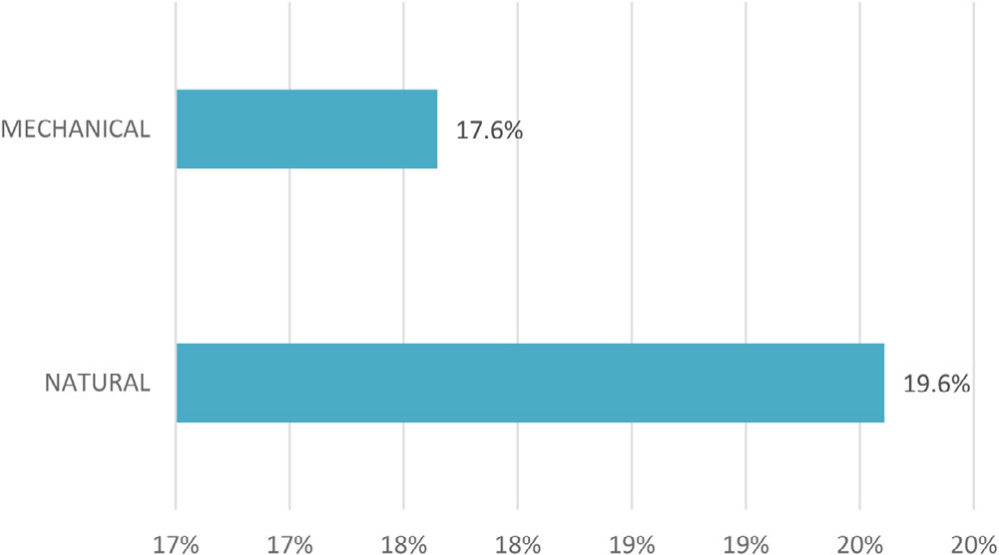
Most commonly used cooling methods in exemplary NZEB buildings across EU [2], [3], [4], [5], [6], [7], [8], [9].

Data linked to this paper allow to visualise NZEBs that use exclusively passive space cooling methods. *Elementary School* and *Kinder-garten Albrechtsberg* in Austria [2] use night ventilation for space cooling while NZEB multi-family house *F3 Brdo*, Ljubljana, uses external shading, manually operated roller blinds [8]. In the collected best practices, there are also NZEBs combining mechanical cooling with natural cooling solutions. *Green Lighthouse* in Denmark uses both heat pumps a well as the night ventilation for cooling [3]. On the other hand, NZEBs, especially in warmer climates, use often mechanical cooling solutions (Fig. 2). The *Leaf House* in Loccioni, Italy uses heat pumps and radiant floors for cooling [6]. The *Technical University Sofia*, Bulgaria uses a VRF system [2].

**Fig. 3:**
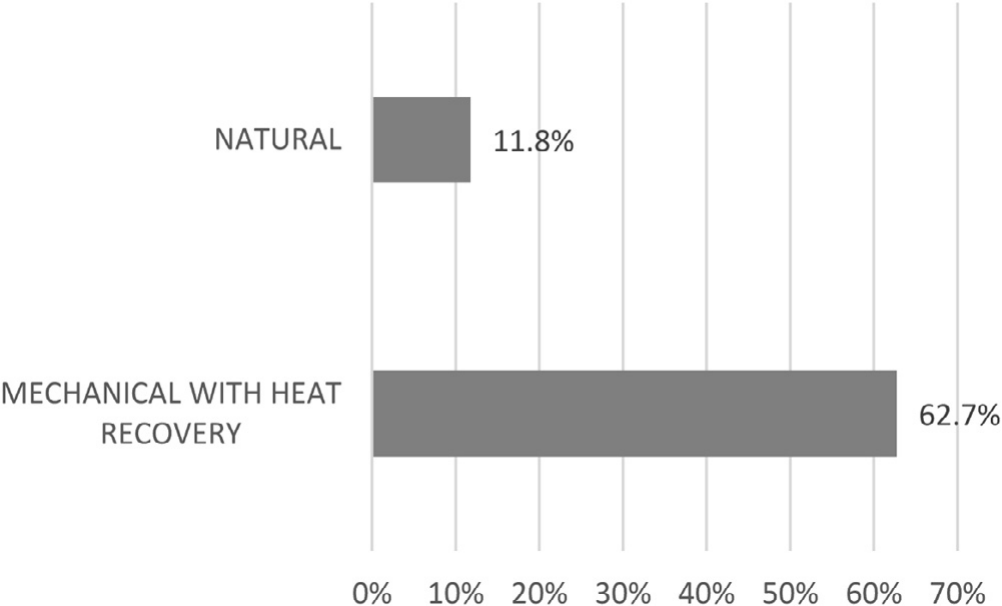
NZEBs having heat recovery system [2], [3], [4], [5], [6], [7], [8], [9].

The majority of the collected buildings with available information about ventilation includes heat recovery systems (Fig. 3). Heat recovery is an air-to-air heat or energy recovery system which works between two sources at different temperatures. Current heat recovery systems are able to recycle about 60–95% of wasted energy, which is promising [13]. In the collected projects best practices, the heat recovery ranges from 70% to 96%. An example of this case is the *École François Mitterand* in France [2] where heat recovery systems are used in winter while natural ventilation is achieved through windows during summer months. The office building in Žminj, Croatia, is ventilated by a forced recuperation ventilation system combined with a heat exchanger [2]. The *Multifunctional School Houthaven* in the Netherlands uses heat recovery units for ventilation [2,3]. The *ENERPOS University complex* in Saint Pierre, France has ceiling fans as well as a VRV system [6].

**Fig. 4:**
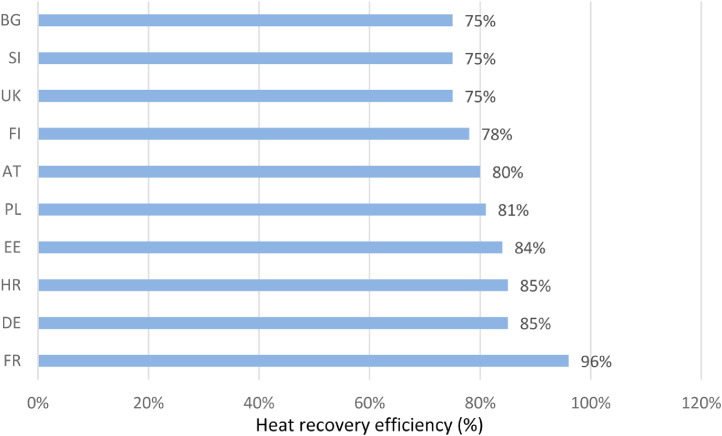
Heat recovery efficiency average in NZEB example buildings per country [2], [3], [4], [5], [6], [7], [8], [9].

In relation to heat recovery efficiency in collected NZEBs best practices (Fig. 4), the *Beddington Zero Energy Development* in London heat recovery system presents an efficiency of 70% [6]. The *Bushbury Hill Primary School* in the United Kingdom has a mechanical ventilation system with heat recovery efficiency by 80% during winter, while it is based on windows during summer [2]. Finally, the *Green Home Nanterre* in France has a decentralised ventilation system of 96% heat recovery [4].

**Fig. 5:**
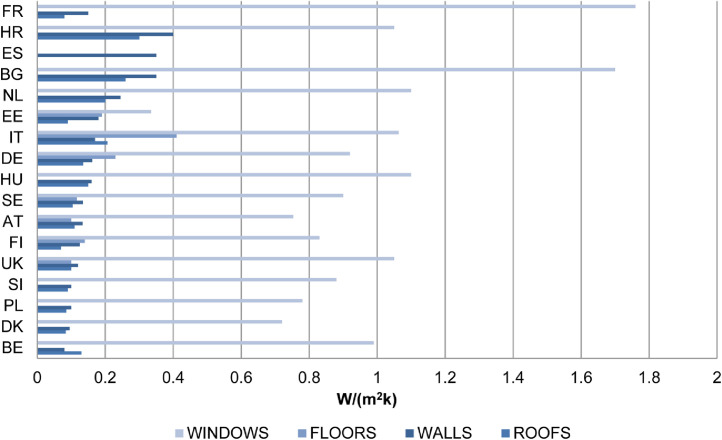
Average U-Values in NZEB example buildings per country [2], [3], [4], [5], [6], [7], [8], [9].

Data are also collected for efficient insulation, an important passive method to reduce energy demand and achieve high energy savings [14,^15^,16]. In Fig. 5, the average U-values per Member State for every building element are presented as collected in NZEBs best practices with available data for walls, roofs, grounds and windows.

U-values range from 0.08 W/m^2^K (NZEB *After School day care center* in Belgium) to 0.4 W/m^2^K (*Faculty of Agriculture, University of Osijek* in Croatia) [2]. The U-values for roofs range from 0.07 W/m^2^K (*Primary School in Budzów* in Poland) to 0.26 W/m^2^K (*Research Centre of the Technical University of Sofia* in Bulgaria) [2]. For floors, U-values range from 0.10 W/m^2^K (*Beddington Zero Energy Development* in London and in *Plus Energy Settlement in Weiz-Gleisdorf* in Austria) to 0.41 W/m^2^K (*Leaf House* in Loccioni in Italy) [6]. For windows, U-values range from 0.27–0.40 W/m^2^K (in *Maardu Day Care Center* in Estonia [9] to 1.76 W/m^2^K (in *École François Mitterand* in France) [2].

Among examples that do not impose the RES requirement in their national NZEB standards, there are the *Primary School Mariagrün* in Austria and the *Bushbury Hill Primary School* in the United Kingdom [3]. Regarding the studied NZEB buildings/projects, 19 out of 51 have available data for their RES contribution. This share ranges from 21% to 105%. The *Plus Energy School Hohen Neuendorf* in Germany uses biomass, CHP and PV to cover the 21% of its energy needs while the *École François Mitterand* in France includes 406 m^2^ of PV to cover an equal share [3]. On the contrary, RES cover the 105% of the primary energy needs of the *Väla Gård* in Sweden (all energy use included, excl. PV generation) [4]. Best practice examples using solar thermal energy for space heating are the *Lehtomäki day care center* in Finland [9] and the *NZEB multi-family house F3 Brdo* in Ljubljana, Slovenia [6]. For example, the *Plus Energy School Hohen Neuendorf* in Germany has 400 m^2^ of photovoltaics [2].

In relation to lighting, *Multifunctional School Houthaven* in the Netherlands uses presence detectors while *Sustainable Building Energy Information Centre Debrecen* in Hungary includes both presence and daylight detectors in its lighting. Best practice examples are the *Luthaa Nursery* in Finland (LED lamps) and the *Childcare Centre* Cologno Monzese in Italy (energy saving lamps) [2,3].

Regarding the costs due to additional NZEB technologies, the lowest ones are identified in *Bushbury Hill Primary School* in the UK, a building based on passive solution for cooling and ventilation in summer [2]. In *Luthaa nursery* in Finland and in *Kleehäuser*, Freiburg in Germany the additional costs are only 10% [2,6]. On the contrary, the additional costs are 50% (or 2600 €/m^2^) in *Green Lighthouse* in Denmark, a building that uses geothermal, solar thermal and PV as renewables, district heating, heat pumps and LED lights [2]. In addition, the impact of NZEB technologies on investment costs in *Väla Gård* is calculated to be around 61% (6% RES, 25% HVAC, 1% DHW, 12% ventilation, 11% heating and 6% windows). The building includes ventilation with heat recovery, ground source heat pumps and PV [4].

**Fig. 6:**
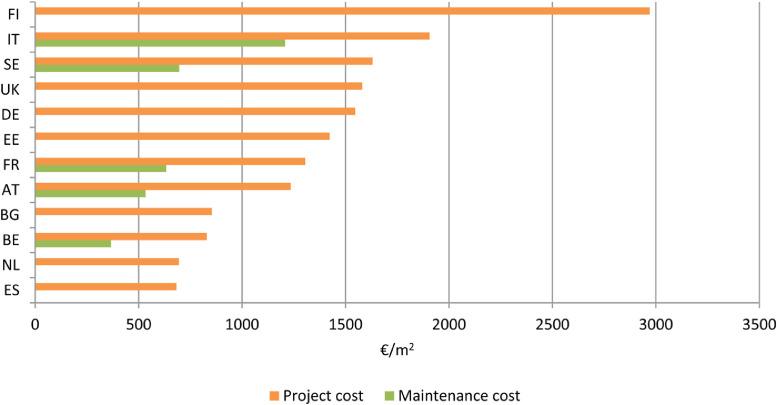
Average project and maintenance cost of best practice example buildings by MS [2], [3], [4], [5], [6], [7], [8], [9].

Fig. 6 shows the average project and lifecycle maintenance costs of collected best practices NZEBs per Member State.

The data from 24 NZEBs show that the project costs range from 682.3 €/m^2^ in *A-32 building, Salburua* in Spain [AZEB, 2018] to 2624 €/m^2^ in *Moretti More* in Italy [4]. Data for maintenance costs are available in 11 buildings/projects from [4]. These data range from 366 €/m^2^ in *Brussels Rings* to 1106 €/m^2^ in *Isola nel Verde* in Italy [4]. The average maintenance costs in these projects are 750 €/m^2^. A broad scale shift towards NZEBs requires an effort in the current market. Cost-effective integration of efficient solutions and renewables remain a major challenge.

## Ethics Statements

No ethics fields involved.

## CRediT authorship contribution statement

**Delia D'Agostino:** Conceptualization, Methodology, Investigation, Writing – original draft, Writing – review & editing. **Sofia Tsemekidi Tzeiranaki:** Data curation, Software, Investigation, Writing – review & editing. **Paolo Zangheri:** Data curation, Investigation. **Paolo Bertoldi:** Supervision.

## Declaration of Competing Interest

The authors declare that they have no known competing financial interests or personal relationships that could have appeared to influence the work reported in this paper.

## Appendix Supplementary materials

Image, application 1
